# Effects of resveratrol supplementation on bone quality: a systematic review and meta-analysis of randomized controlled trials

**DOI:** 10.1186/s12906-021-03381-4

**Published:** 2021-08-22

**Authors:** Qiangqiang Li, Guangpu Yang, Hongtao Xu, Shaowen Tang, Wayne Yuk-wai Lee

**Affiliations:** 1 State Key Laboratory of Pharmaceutical Biotechnology, Division of Sports Medicine and Adult Reconstructive Surgery, Department of Orthopedic Surgery, Nanjing Drum Tower Hospital, The Affiliated Hospital of Nanjing University Medical School, 321 Zhongshan Road, Nanjing, Jiangsu 210008 China; 2 SH Ho Scoliosis Research Laboratory, Joint Scoliosis Research Centre of the Chinese University of Hong Kong and Nanjing University, Faculty of Medicine, The Chinese University of Hong Kong, Hong Kong, China; 3 Li Ka Shing Institute of Health Sciences, The Chinese University of Hong Kong, Hong Kong, China; 4 Department of Orthopaedics and Traumatology, Faculty of Medicine, The Chinese University of Hong Kong, Hong Kong, China; 5 Department of Epidemiology, School of Public Health, Nanjing Medical University, Nanjing, 211166 China

**Keywords:** Resveratrol, Bone mineral density, Bone biomarkers

## Abstract

**Background:**

The results from clinical trials have revealed that the effects of resveratrol supplementation on bone mineral density (BMD) and bone biomarkers are inconsistent. Our objective was to determine the effects of resveratrol supplementation on BMD and serum bone biomarkers.

**Methods:**

PubMed, Cochrane library, EMBASE, Web of science and Scopus were searched up to August 24, 2020. Two reviewers independently performed the articles search and screen according to defined selection criteria. The study quality of the randomized controlled trials (RCTs) was evaluated with the Cochrane scoring system. Heterogeneity among studies was examined by Cochrane Q test. Retrieved data were pooled after mean differences (MD) were computed between two groups for BMD and serum biomarkers. Subgroup analyses were performed to evaluate a potential difference in terms of dose of resveratrol and intervention duration. Sensitivity analysis was executed by omitting studies with imputed values in order to evaluate the influence of these studies on the overall results.

**Results:**

Ten eligible studies involving 698 subjects were included in this meta-analysis with 401 participants receiving resveratrol and 297 receiving placebo. Supplementation of resveratrol had no statistically significant effects on areal bone mineral density (aBMD) at lumbar spine (MD: -0.02, 95% CI: − 0.05, 0.01, *p* = 0.26, I^2^ = 6%), total hip BMD (MD: -0.01, 95% CI: − 0.04, 0.02, *p* = 0.65, I^2^ = 0%), and whole body BMD (MD: 0.00, 95% CI: − 0.02, 0.02, *p* = 0.74, I^2^ = 0%). Supplementation of resveratrol also did not result in significant change in bone serum markers, including serum alkaline phosphatase (ALP), bone alkaline phosphatase (BAP), osteocalcin (OCN), procollagen I N-terminal propeptide (PINP), C-terminal telopeptide of type I collagen (CTX) and parathyroid hormone (PTH). Subgroup analysis showed the effect of resveratrol supplementation on BMD and serum bone markers were similar in trails of different doses, intervention duration, and pathological conditions of the participants.

**Conclusion:**

Resveratrol supplementation did not show any significant effect on BMD or serum bone markers with the current evidence. Further investigation with more well-organized multicentre randomized trial is warranted.

**Supplementary Information:**

The online version contains supplementary material available at 10.1186/s12906-021-03381-4.

## Background

Osteoporosis is a skeletal disorder characterized by low bone mass, structural deterioration, decreased bone strength, and increased risk of fractures [1]. Osteoporosis has become one of the major challenging world-wide public health problems particularly in ageing societies [2, 3]. Low bone mineral density (BMD) is a major risk factor for osteoporotic fracture, and it has been considered as a surrogate endpoint for fracture risk [4]. Current pharmacologic drugs that are used to treat osteoporosis mainly aim to reduce excessive bone resorption (e.g. estrogen and bisphosphonates) or promote bone formation (e.g. parathyroid hormone (PTH)), and to a lesser degree a combination of both (e.g. anti-sclerostin antibody) [5]. However, there is growing concern about the long-term use of these drugs due to their off-target effects [6–9]. Therefore, there is a clear demand of continuing efforts in research and development of safer preventative and/or therapeutic agents.

Resveratrol (3,5,4′-trihydroxystilbene) belongs to a family of polyphenolic compounds known as stilbenes found in nuts, grapes and other plant sources [10], which has shown to be beneficial for age-related degenerative diseases such as type 2 diabetes and cardiovascular disease for its properties of antioxidant, anti-inflammatory, anti-carcinogenic, improving endothelial function and mimicking calorie restriction [11]. Many preclinical studies have shown the protective effects of resveratrol exist in bone tissue in different animal models of osteoporosis [12–14]. The molecular mechanisms underlying the anti-osteoporotic effects of resveratrol were associated with its positive effect on osteogenesis and bone formation [15, 16], inhibitory effect on osteoclastogenesis and bone resorption [17, 18], antioxidative effect on bone cells [19, 20], and promoting effect on the osteogenic differentiation of bone mesenchymal stem cells [21, 22]. Therefore, resveratrol offers the promise of being an effective therapeutic target for osteoporosis through multiple actions on both osteoblasts and osteoclasts [1].

Despite the abovementioned preclinical evidence, randomized controlled trials of resveratrol supplementation on bone are explorative and show controversy [23–25], and therefore the effectiveness of resveratrol supplementation for improving bone quality is unclear. A previous systematic review and meta-analysis compared bone biomarkers in subjects who received resveratrol or placebo. The results of the study showed a significant increase in serum alkaline phosphatase (ALP) and bone alkaline phosphatase (BAP) values after resveratrol treatment compared with placebo [26]. However, bone biomarkers only partially reflect the process of bone remodeling instead of bone quality outcome. Considering BMD is a surrogate endpoint for fracture risk that allows exploration of biological effects in clinical trials, and the effect of resveratrol supplementation on BMD has not been evaluated by meta-analysis till now. Therefore, for the first time, we aimed to evaluate the effect of resveratrol supplementation on BMD and bone biomarkers through a systematic review and meta-analysis of available randomized clinical trials (RCTs).

## Methods

For this review, we followed the preferred reporting items for systematic reviews and meta-analyses (PRISMA) statement [27].

### Search strategy

**Fig. 1:**
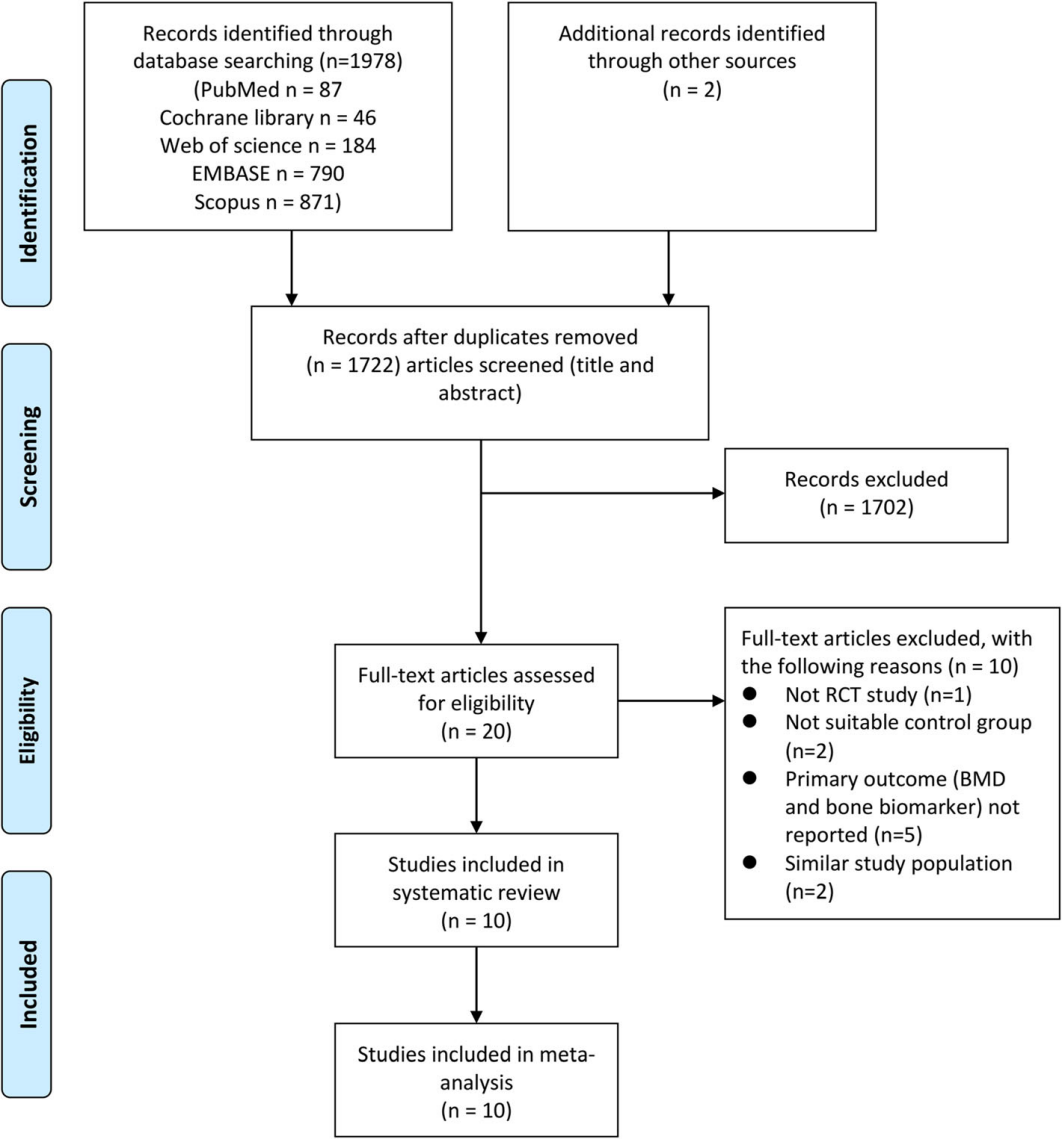
Flow diagram of the inclusion process

PubMed, Cochrane library, EMBASE, Web of science and Scopus were searched to retrieve relevant papers dating up to August 24, 2020 with no language restriction. Our search strategy was based on a PICOS methodology and both Medical Subject Headings (MeSh) and text words were used (supplementary Table 1). Literature search strategies were developed using terms which were related to resveratrol, bone density and bone biomarkers. Resveratrol related terms included “resveratrol” or “3,5,4′-trihydroxystilbene” or “4′,5-trihydroxystilbene” or “3,4′,5-stilbenetriol” or “trans-resveratrol-3-O-sulfate” or “trans resveratrol 3 O sulfate” or “SRT 501” or “SRT501” or “SRT-501” or “501–36-0” or “cis-resveratrol” or “cis resveratrol” or “trans-resveratrol” or “trans resveratrol” or “resveratrol-3-sulfate” or “resveratrol 3 sulfate”. Bone density related terms included “bone and bones” or “bone microarchitecture” or “bone geometry” or “bone density” or “bone mineral density” or “BMD” or “bone mass density” or “bone mineral content” or “BMC”. Bone biomarkers related terms included “bone biomarkers” or “bone turnover” or “bone metabolism” or “alkaline phosphatase” or “ALP” or “bone alkaline phosphatase” or “BAP” or “calcium” or “Ca” or “parathyroid hormone” or “PTH” or “procollagen I N-terminal propeptide” or “P1NP” or “C-terminal telopeptide of type I collagen” or “CTX” or “osteocalcin” or “OCN” or “N-telopeptide” or “NTX” or “N-telopeptide of type I collagen”. The reference lists of available studies were manually searched to identify additional articles for potential inclusions. The selection process was conducted by two individual investigators (QQL and GPY) independently and disagreements were resolved through discussions. A flow diagram of our search strategy is depicted in Fig. 1.

### Literature inclusion and exclusion criteria

Two reviewers (QQL and GPY), working independently and in duplicate, identified and evaluated potentially eligible trials according to predefined inclusion criteria. Inclusion and exclusion criteria were as follows: (1) Participants: adult participants (> = 18 years old) regardless of their sex or pathological conditions. However, we excluded studies on non-human subjects, pregnant or lactating females. (2) Interventions: Resveratrol alone or resveratrol combined with other routine drugs (like antihypertensive drugs) were considered as interventions. There were no restrictions on the administration method of resveratrol. However, we excluded studies in which extra intervention like hormone replacement therapy, bisphosphonates, and other drugs affecting bone metabolism were taken along with resveratrol. (3) Controls: If resveratrol was administrated only, then the control group should receive placebo only; if resveratrol was administrated as an adjunct to another drug or supplement, the control group had to receive the same drug or supplement plus placebo. We excluded studies in which the control group underwent additional therapies compared to intervention group, making it impossible to investigate the effects of resveratrol alone. (4) Outcomes: The primary outcome measures were aBMD of total body, femoral neck, lumbar spine (L1-L4 or L2-L4), and whole hip by dual-energy X-ray absorptiometry (DXA). The secondary outcome measures were bone biomarkers (ALP, BAP, PTH, osteocalcin (OCN), C-terminal telopeptide of type I collagen (CTX), N-terminal telopeptide of type I collagen (NTX), and procollagen I N-terminal propeptide (PINP)). (5) Study design: We included RCTs performed in human (either parallel or crossover designs). Disagreements regarding the study selection process were resolved by discussion with the third researcher (HTX).

### Data extraction

QQL extracted data from each eligible trial according to prepared data extraction form (Table 1 and Table 2). The extracted data was checked by another investigator (GPY) to reduce reviewer errors. If there were discrepancies, group consensus and a third reviewer was consulted to ensure accuracy of data. Data extracted from the eligible studies were: first author, year of publication, location of study, study population, characteristics of participants (age, sex), number of participants in each group, loss to follow up, compliance, study duration and the final results of resveratrol supplementation comparisons with the control group, daily dose of resveratrol, route of administration, form of resveratrol, brand of resveratrol, purity of resveratrol, and any reported adverse events. When the intermediary results of the clinical studies were reported at different time points of the study, only the final data at the end of the intervention period were considered for this review.

**Table 1:** Demographic characteristics of the included studies

First author (Year)	Country	Study population	Gender	Mean age (year)	Sample size	Loss to-follow up	Compliance	Study duration	Findings
Wong et al. (2020)	Australia	post menopause women	F	Placebo: 65.8 ± 1.3 Resv: 64.3 ± 1.3	Placebo: 65 Resv: 63	Placebo: 11% Resv: 14%	Placebo: 95% Resv: 94%	12 months	12 months supplementation with resveratrol increases BMD in lumbar spine and neck of femur in postmenopausal women.
Ornstrup et al. (2014)	Denmark	obese men with metabolic syndrome	M	Placebo: 48.2 ± 6.4 Low dose: 48.9 ± 6.5 High dose: 50.9 ± 5.9	Placebo: 24 Low dose: 21 High dose: 21	Placebo: 8% Low dose: 9% High dose: 16%	Placebo: 97% Low dose: 93% High dose: 96%	16 weeks	High dose resveratrol supplementation increases serum BAP level and vBMD in lumber spine in a dose-dependent manner in obese men.
Bo et al. (2018)	Italy	type 2 diabetes patients	F/M	Placebo: 65.4 ± 8.8 Low dose: 64.9 ± 8.6 High dose: 65.0 ± 7.6	Placebo: 58 Low dose: 59 High dose: 62	Placebo: 6% Low dose: 9% High dose: 5%	> 95% overall	6 months	6 months supplementation of 500 mg resveratrol increases whole body BMD and BMC and serum ALP in type 2 diabetes.
Poulsen et al. (2014)	Denmark	obese nondiabetic men	M	Placebo: 31.9 ± 2.9 Resv: 44.7 ± 3.5	Placebo: 12 Resv: 12	Placebo: 0% Resv: 1%	Placebo: 89.2% Resv: 88.9%	4 weeks	Short term supplementation of resveratrol increases plasma level of BAP in obese non-diabetic men but not other bone markers.
Heebøll et al. (2016)	Denmark	non-alcoholic fatty liver disease	F/M	*Placebo: 43.5 (21–69) Resv: 43.2 (22–67)	Placebo: 13 Resv: 13	Placebo: 0% Resv: 1%	Placebo: 97% Resv: 81%	6 months	6 months supplementation of resveratrol did not significantly increase serum ALP compared with placebo.
Anton et al. (2014)	USA	healthy elderly	F/M	Placebo: 73.3 ± 2.06 Low dose: 73.17 ± 2.08 High dose: 73.60 ± 2.53	Placebo: 10 Low dose: 12 High dose: 10	Placebo: 17% Low dose: 14% High dose: 23%	Placebo: 93% Low dose: 93% High dose: 93%	3 months	12 weeks supplementation of high-dose resveratrol significantly increased serum ALP compared with placebo but not Ca.
Movahed et al. (2013)	Iran	type 2 diabetic patients	F/M	Placebo: 51.81 ± 6.99 Resv: 52.45 ± 6.18	Placebo: 31 Resv: 33	Placebo: 0% Resv: 3%	unclear	6 weeks	6 weeks supplementation of resveratrol did not significantly increase serum ALP compared with placebo.
Asghari et al. (2018)	Iran	non-alcoholic fatty liver disease	F/M	*Placebo: 38.50 (30, 48) Resv: 40.00 (22, 58)	Placebo: 26 Resv: 25	Placebo: 13% Resv: 17%	over 90% overall	12 weeks	12 weeks supplementation of resveratrol did not significantly increase serum ALP compared with placebo.
Tomé-Carneiro et al. (2013)	Spain	type 2 diabetes and hypertensive patients with coronary artery disease	M	GE placebo: 60 ± 10 GE with resv: 63 ± 12	GE placebo: 13 GE with resv: 13	GE placebo: 0% GE with resv: 0%	> 95%	12 months	12 months supplementation with resveratrol significantly reduced serum ALP.
van der Made et al. (2015)	Italy	overweight and obese subjects	F/M	Overall: 60 ± 7	Placebo: 22 Resv: 23	GE placebo: 12% GE with resv: 8%	99%	4 weeks	4 weeks supplementation with resveratrol significantly increased serum ALP compared with placebo.

Data are reported as mean ± standard deviation. *expressed as median and range. Abbreviation: Resv, resveratrol; BMD, bone mineral density; BAP, bone alkaline phosphatase; CTX, C-terminal telopeptide of type I collagen; vBMD, volumetric bone mineral density; BMC, bone mineral content; ALP, alkaline phosphatase; GE, grape extract

**Table 2:** Comparison of resveratrol intervention among included studies

First author (Year)	Identical with placebo	Dosage and frequency	Total dosage/daily	Route of administration	Administration form	Alone or in combination with other medications	Company	Purity	Adverse events
Wong et al. (2020)	Yes	75 mg twice daily (morning and evening)	150 mg	Oral	Capsule	Alone	Evolva SA (Reinach, Switzerland)	> 98% tans-resveratrol	Not related to resveratrol supplementation
Ornstrup et al. (2014)	Unclear	500 mg twice daily and 75 mg twice daily	1000 mg and 150 mg	Oral	Tablet	Alone	Resv: Evolva SA (Reinach, Switzerland) Placebo: Robinson Pharma (Santa Ana, CA, USA)	> 98% tans-resveratrol	One case of transient pruritic skin rash
Bo et al. (2018)	Year	500 mg/daily and 40 mg/daily (morning)	500 mg and 40 mg	Oral	Capsule	Hypoglycemic treatment	Biotivia Bioceuticals (International SrL, Italy)	99.7% and a 97.9% purity of trans-resveratrol	No serious adverse event
Poulsen et al. (2014)	Unclear	500 mg three times daily (morning)	1500 mg	Oral	Tablet	Alone	Resv: Fluxome (Stenlose, Denmark) Placebo: Robinson Pharma (Santa Ana, CA, USA)	Unclear	The rate is not significantly different from placebo
Heebøll et al. (2016)	Yes	500 mg three times daily	1500 mg	Oral	Capsule	Alone	Unclear	Unclear	Two serious cases: gastrointestinal side effects and febrile leukopenia and thrombocytopenia
Anton et al. (2014)	Unclear	150 mg twice daily and 500 mg twice daily (morning and evening)	1000 mg and 300 mg	Oral	Capsule	Alone	Reserveage Organics	within 7% of the stated mg dose	The rate is not significantly different from placebo
Movahed et al. (2013)	Unclear	500 mg twice daily	1000 mg	Oral	Capsule	Antidiabetic medications	Resv: Biotivia, Bioceuticals International SrI, (Italy) Placebo: Biotivia, Bioceuticals International SrI, (Italy)	99%	No serious adverse event
Asghari et al. (2018)	Yes	300 mg twice daily	600 mg	Oral	Capsule	Alone	Unclear	Unclear	No serious adverse event
Tomé-Carneiro et al. (2013)	Yes	8 mg for the first 6 months and 16 mg for the following 6 months	8 mg for the first 6 months 16 mg for the following 6 months	Oral	Capsule	Statins, β-blockers, antiplatelets, RAS-blockers treatment and oral antidiabetics	Laboratorios Actafarma S.L. (Pozuelo de Alarcón, Spain)	Unclear	No adverse event
van der Made et al. (2015)	Yes	75 mg twice daily	150 mg	Oral	Capsule	Antidepressants, antihypertensives or antacids	DSM Nutritional Products Ltd. (Kaiseraugst, Switzerland)	99.9% trans-resveratrol	No adverse event

Abbreviation:* Resv* resveratrol; *RAS* renin-angiotensin system

We used endpoint data rather than change data from baseline to maximise data availability considering most of the included studies (8 out of 10 studies) did not report the change data from baseline and the standard deviation (SD). In addition, the comparison of final measurements in a randomized trial in theory estimates the same quantity as the comparison of changes from baseline [28]. For the single study in which BMD outcomes were presented as percentage change from baseline [23], and no endpoint data were available, we imputed endpoint data using the baseline BMD and percentage change from baseline and the SD of the baseline data for the endpoint SD [29]. Where studies reported absolute change from baseline and endpoint data were not available [30], we imputed endpoints using baseline plus change for the mean and using the SD of the baseline data for the endpoint SD. If the data were only reported as graph, we extracted the values using GetData Graph Digitizer 2.24 software. If studies reported the median, range and the sample size, then the mean and SD were estimated [31, 32]. If studies reported standard error of the mean (SEM) only, then the SD was estimated as follows: SD = SEM × square root (n), being n the subjects’ number [28]. If studies reported the mean, 95% CI and the sample size, then the mean and SD were estimated according to the Cochrane Handbook [28]. For studies with more than one resveratrol dosage group, we divided the number in the control groups by the number of the treatment arms to avoid double-counting problem [28].

### Risk of bias assessment

Two reviewers (QQL and GPY), working independently and in duplicate, evaluated the quality of the eligible studies using the Cochrane scoring system [33] of 7 points based on the following criteria: (1) Random sequence generation, (2) Allocation concealment, (3) Blinding of participants and personnel, (4) Blinding of outcome assessment, (5) Incomplete outcome data, (6) Selective reporting, (7) Other sources of biases such as baseline imbalance. Based on the recommendations of the Cochrane Handbook, risk of bias was judged to be L, H, and U, which is interpreted as low risk, high risk, and unknown risk of bias respectively.

### Subgroup analysis

Subgroup analyses were performed by the dose of resveratrol and intervention period to determine whether the effects of supplementation varied by these factors. As the number of studies were small, the cut-off of dose of 500 mg daily and duration of 3 months for resveratrol were chosen on the basis of the sufficient data available at this cut-off to allow for subgroup analysis.

### Statistical analysis

Meta-analysis was conducted by combining studies which were clinically similar in participants, intervention, comparator and outcome (PICO). All analyses were carried out using Review Manager 5.1 (Cochrane Collaboration, UK). Effect size were expressed as mean differences (MDs) and 95% confidence intervals (CI) with forest plots. Heterogeneity among the included studies was quantitatively assessed with the Chi^2^ test (*p* value < 0.1) and I^2^ test, with I^2^ > 50% indicating significant heterogeneity [34]. We used a random-effects model if a significant heterogeneity was detected; otherwise, a fixed-effects model was applied. Studies containing different groups of resveratrol were independently entered. Funnel plots were not included in this study as tests for funnel plot asymmetry is not recommended when a meta-analysis contains fewer than 10 studies, due to the low power for detecting true effects not ascribed to chances [33]. We performed a sensitivity analysis by omitting studies for which data were imputed.

## Results

### Selection and identification of studies

Figure 1 shows the flow diagram of paper inclusion and selection process. In summary, a total of 1978 publications were identified from the following databases including PubMed (87), Cochrane library (46), EMBASE (790), Web of science (184), and Scopus (871), which yielded 1978 papers after removing duplicates (258 articles). Two additional relevant papers were recognized by searching the reference list of eligible publications. After screening the retrieved manuscripts based on titles and abstracts, we retrieved 20 full texts [23–25, 35] [30, 36–38] [39–41] [42, 43] [44, 45] [46–48] [49]. 10 were excluded [41–50] and 10 were finally included in the review and meta-analysis according to inclusion and exclusion criteria [23–25, 30, 35–40]. Trials were excluded for the following reasons: not randomized controlled trials [47] (*n* = 1), unsuitable control group [45, 46] (*n* = 2), outcome of interest not reported [41–44, 50] (*n* = 5), and outcomes from the same dataset [48, 49] (n = 2).

### Study characteristics

Table 1 describes the baseline characteristics of the included trials. Overall, 23 treatment arms were extracted from 10 RCTs that included a total of 698 participants, of which 401 participants were in the resveratrol group and 297 were in the placebo group. The year of publication of the included trials ranged from 2013 to 2020. Three trials were conducted in Denmark [23, 35, 36], two in Iran [37, 38], one in the United State [30], one in Australia [25], one in Spain [39], and two in Italy [24, 40]. Three studies were conducted on type 2 diabetes patients [24, 37, 39], two on patients with non-alcoholic fatty liver disease (NAFLD) [36, 38], three on obese population [23, 35, 40], one on healthy elderly [30] and one on postmenopausal women [25].

Table 2 describes resveratrol intervention methods among the trials. The daily dose of resveratrol ranged from 8 mg to 1500 mg, and the intervention periods ranged form 4 weeks to 12 months. All resveratrol supplements and placebos were in the form of capsules or tablets and were administrated orally either alone (six in ten studies) or in combination with other medications including antidiabetic, antiplatelets, antacids medications etc. (four in ten studies).

Three of ten studies reported pre to post changes in BMD at lumbar spine, total hip and whole body [23] [24] [25], and all studies measured changes in serum bone biomarkers [23–25, 30, 35, 36] [37–40], with three trials reporting serum OCN [23, 25] [35], nine trials reporting ALP [23, 24, 35, 36] [30, 37–39] [40], two trials reporting PINP [23] [35], three trials reporting CTX [23, 25, 35], two trials reporting BAP [23, 35] and two trials reporting PTH [23, 35]. The three trails reporting BMD had a total of 386 participants (*n* = 235 in the resveratrol group and *n* = 151 in the placebo group).

### Risk of bias

**Fig. 2:**
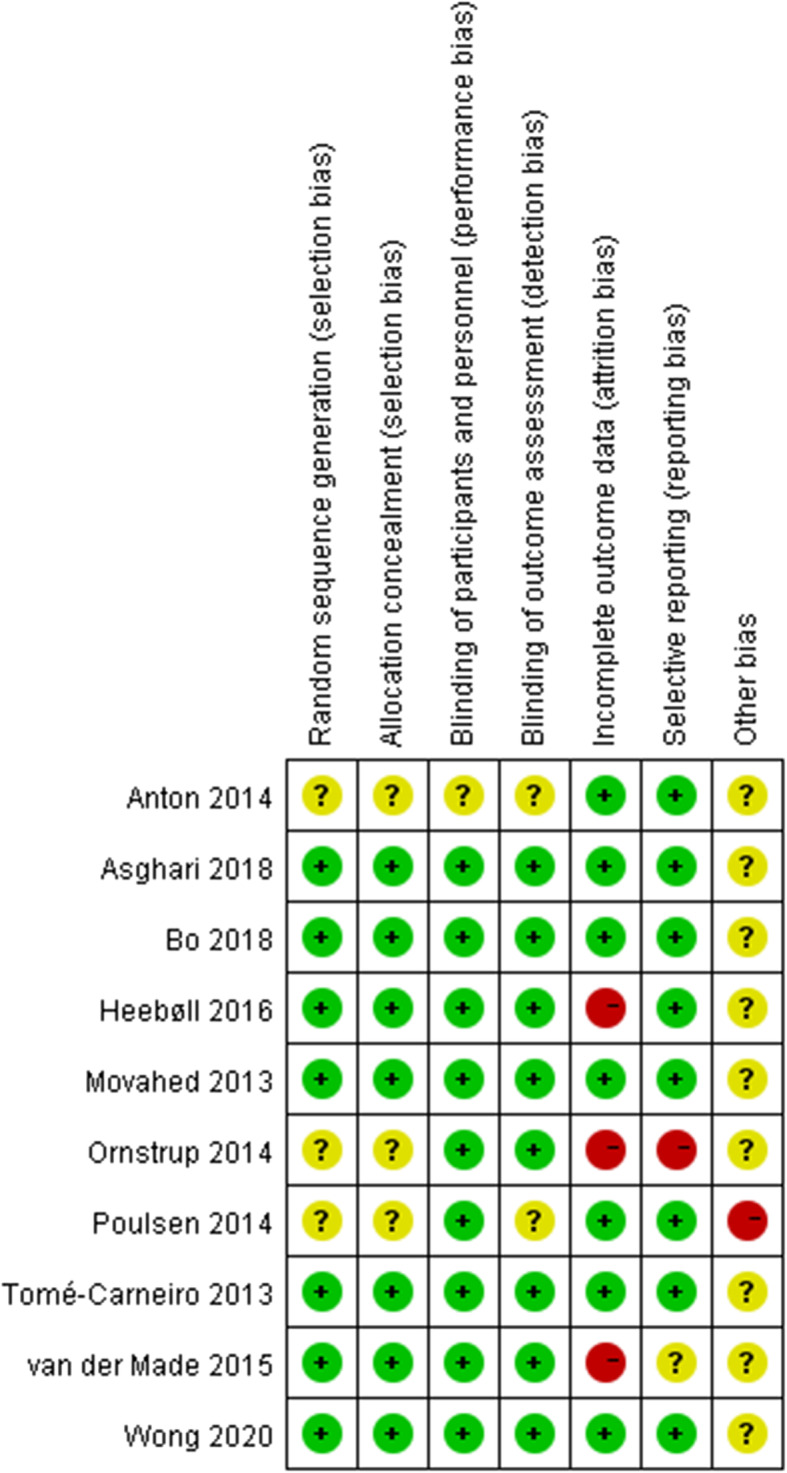
Risk of bias assessment of the included studies. +, low risk of bias; −, high risk of bias; ?, unclear risk of bias

The risk of bias assessment was displayed in Fig. 2. Overall, the level of evidence of the included studies was high, with seven of ten studies considered to have a low risk of bias for random sequence generation/allocation, blinding of participants, blinding of outcome assessment [24, 25, 36–40]. Three studies were considered to have unclear risk of bias for concealment and blinding procedures due to insufficient information of the procedures [23, 30, 35]. Three studies were rated high risk of bias for incomplete outcome data because they did not report the data of the final assessment [23, 36, 40]. One study was rated high risk of other sources of bias due to baseline imbalance [35].

### Effect of resveratrol supplementation on BMD

**Fig. 3:**
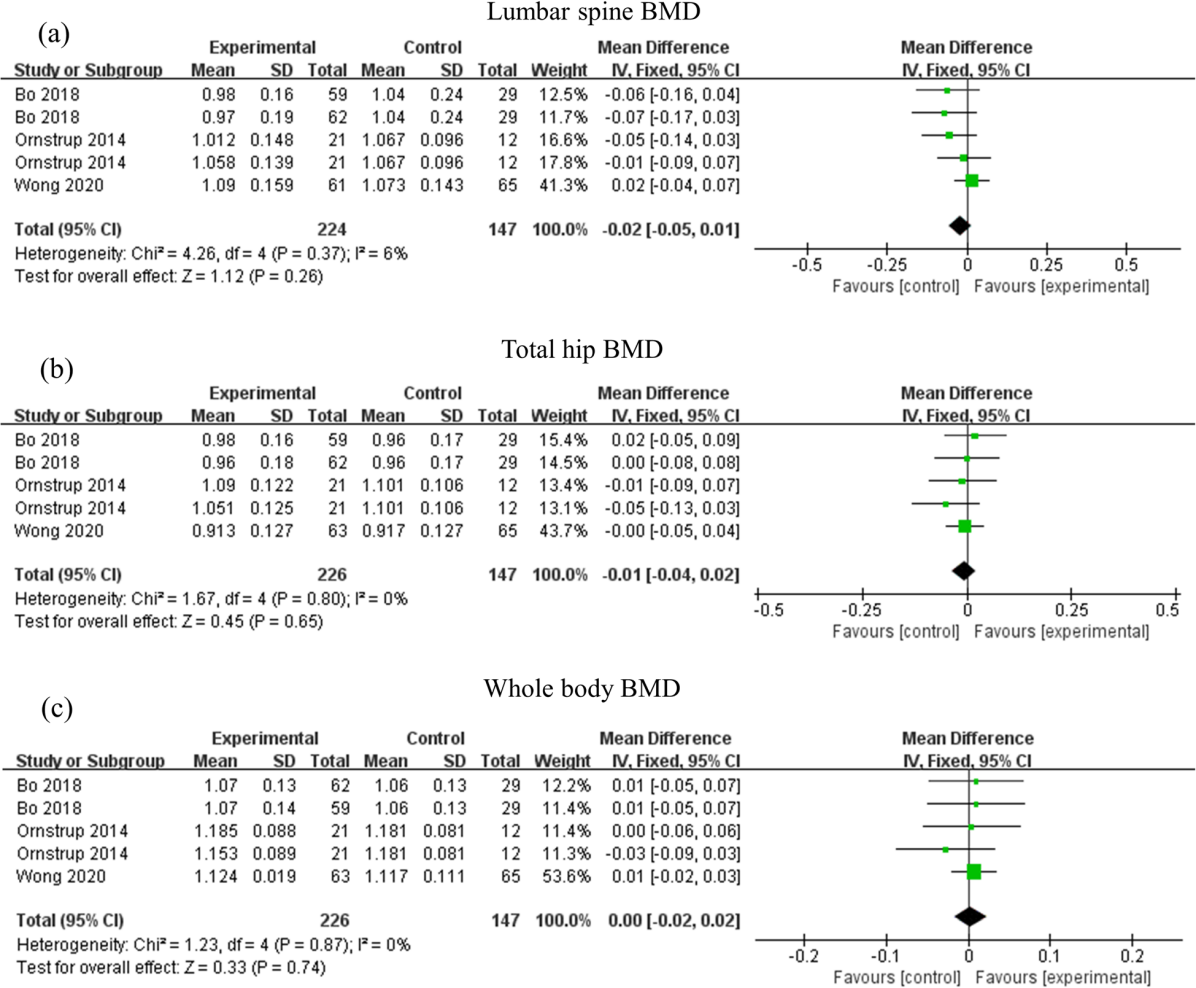
Effects of resveratrol supplementation on bone mineral density. The funnel plots of main effects of resveratrol supplementation on BMD of (a) lumbar spine, (b) total hip, and (c) whole body. Abbreviations: BMD, bone mineral density

A total of three studies compared placebo with resveratrol on BMD [23–25]. As shown in Fig. 3 and Table 3, the results suggested resveratrol supplementation ranging from 16 weeks to 12 months did not have statistically significant effects on the change in lumbar spine BMD (MD: -0.02, 95% CI: − 0.05, 0.01, *p* = 0.26, 3 trials, 370 patients) and (I^2^ = 6%, *p* = 0.37), total hip BMD (MD: -0.01, 95% CI: − 0.04, 0.02, *p* = 0.65, 3 trials, 373 patients) and (I^2^ = 0%, *p* = 0.80), and whole body BMD (MD: 0.00, 95% CI: − 0.02, 0.02, *p* = 0.74, 3 trials, 373 patients) and (I^2^ = 0%, *p* = 0.87). The sensitivity analysis omitting studies that used imputed data also did not significantly affect the results (supplementary Tables 2, 3 and 4).

**Table 3:** Summary of finding table of resveratrol compared to placebo for bone mineral density

Resveratrol compared to Placebo for bone mineral density in human				
Patient or population: Adults Setting: Intervention: Resveratrol Comparison: Placebo				
**Outcome** **№ of participants** **(studies)**	**Anticipated absolute effects (95% CI)**			**Certainty**
	**Without resveratrol**	**With resveratrol**	**Difference**	
BMD at lumbar spine assessed with: dual-energy X-ray absorptiometryfollow up: range 16 weeks to 12 months № of participants: 370 (3 RCTs)	The mean BMD at lumbar spine was 1.04–1.073	–	MD 0.02 lower (0.05 lower to 0.01 higher)	⨁◯◯◯ VERY LOW ^a,b,c^
Serum PINP follow up: range 4 weeks to 16 weeks № of participants: 90 (2 RCTs)	The mean serum PINP ranged from 8.6–41.3	–	MD 2.92 lower (6.33 lower to 0.5 higher)	⨁◯◯◯ VERY LOW ^a,b,d^

*The risk in the intervention group (and its 95% confidence interval) is based on the assumed risk in the comparison group and the relative effect of the intervention (and its 95% CI)

CI: Confidence interval; MD: Mean difference

**GRADE Working Group grades of evidence**

High certainty: We are very confident that the true effect lies close to that of the estimate of the effectModerate certainty: We are moderately confident in the effect estimate: The true effect is likely to be close to the estimate of the effect, but there is a possibility that it is substantially different

Low certainty: Our confidence in the effect estimate is limited: The true effect may be substantially different from the estimate of the effectVery low certainty: We have very little confidence in the effect estimate: The true effect is likely to be substantially different from the estimate of effect

**Explanations**

a. There were some high risk of bias of included trials according to the risk of bias assessment

b. There were some incomplete outcome data among the included studies and some data were imputed for the analysis

c. There were only three studies showing positive or no effect, it seems that studies showing negative effect have not been published

d. Results were based on two studies with a small sample size and wide 95% confidence interval

### Effect of resveratrol supplementation on bone biomarkers

**Fig. 4:**
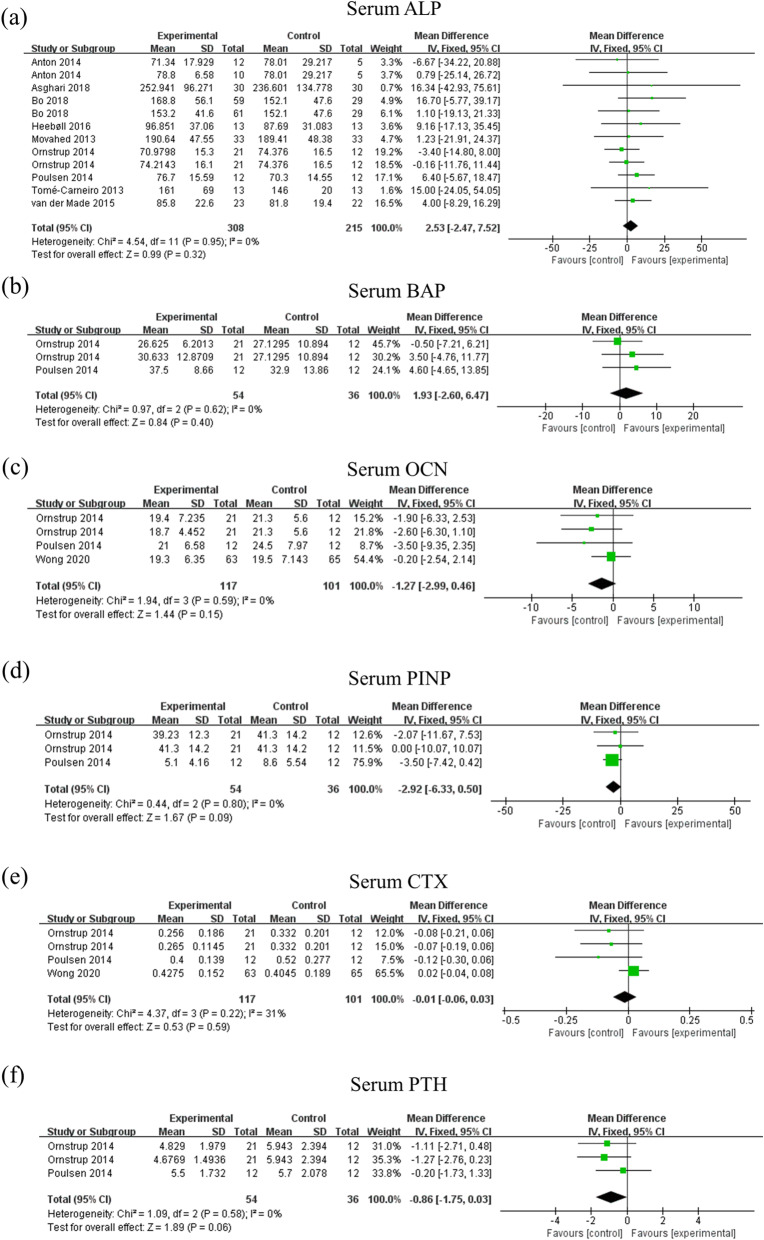
Effects of resveratrol supplementation on serum bone biomarkers. The funnel plots of main effects of resveratrol supplementation on (a) serum ALP, (b) serum BAP, (c) serum OCN, (d) serum PINP, (e) serum CTX, (f) serum PTH. Abbreviations: ALP, alkaline phosphatase; BAP, bone alkaline phosphatase; OCN, osteocalcin; PINP, procollagen I N-terminal propeptide; CTX, C-terminal telopeptide of type I collagen; PTH, parathyroid hormone

As shown in Fig. 4 and Table 3, there were no significant differences between placebo and resveratrol group in the serum biomarkers including ALP (MD: 2.53, 95% CI: − 2.47, 7.52, *p* = 0.32, 9 studies, 523 participants), BAP (MD: 1.93, 95% CI: − 2.60, 6.47, *p* = 0.40, 2 studies, 90 participants), CTX (MD: -0.01, 95% CI: − 0.06, 0.03, *p* = 0.59, 3 studies, 218 participants), OCN (MD: -1.27, 95% CI: − 2.99, 0.46, *p* = 0.15, 3 studies, 218 participants), PINP (MD: -2.92, 95% CI: − 6.33, 0.50, *p* = 0.09, 2 studies, 90 participants), and PTH (MD: -0.86, 95% CI: − 1.75, 0.03, *p* = 0.06, 2 studies, 90 participants). The intervention duration of the above analysis ranged from 4 weeks to 12 months. There was no significant statistical heterogeneity between studies of the above outcomes. The sensitivity analysis suggested that the effect of resveratrol on serum ALP remains unchanged after omitting the studies with imputed values (supplementary Table 5).

**Fig. 5:**
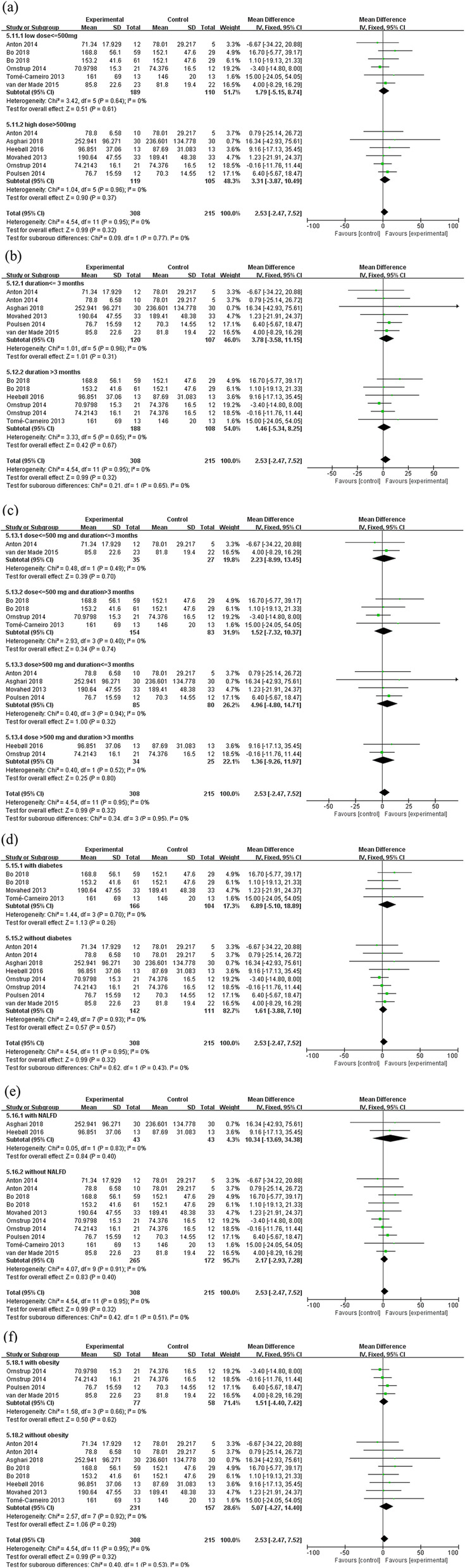
Subgroup analysis of resveratrol supplements versus placebo on serum ALP. The funnel plots of subgroup analysis of the effects of (a) doses of resveratrol, (b) intervention duration, (c) doses and intervention duration, (d) with or without diabetes, (d) with or without NAFLD, and (e) with or without obesity on serum ALP. Abbreviations: ALP, alkaline phosphatase; NAFLD, non-alcoholic fatty liver disease

When the studies were categorized according to resveratrol administered dose, the effects of resveratrol on serum ALP were comparable between subsets of studies with ≤500 mg/day (MD: 1.79, 95% CI: − 5.15, 8.74, *p* = 0.61) or > 500 mg/day (MD: 3.31, 95% CI: − 3.87, 10.49, *p* = 0.37) (Fig. 5a). With respect to intervention duration (≤ or > 3 months), no significant change in serum ALP was observed between subsets of trials lasting less than 3 months (MD: 3.78, 95% CI: − 3.58, 11.15, *p* = 0.31) or above (MD: 1.46, 5% CI: − 5.34, 8.25, *p* = 0.67) (Fig. 5b). With respect to both resveratrol dose and intervention duration, the effects of resveratrol on serum ALP were still comparable between subsets of studies with ≤500 mg/day and ≤ 3 months (MD: 2.23, 95% CI: − 8.99, 13.45, *p* = 0.70), ≤ 500 mg/day and > 3 months (MD: 1.52, 95% CI: − 7.32, 10.37, *p* = 0.74), > 500 mg/day and ≤ 3 months (MD: 4.96, 95% CI: − 4.80, 14.71, *p* = 0.32), or > 500 mg/day and > 3 months (MD: 1.36, 95% CI: − 9.26, 11.97, *p* = 0.80) (Fig. 5c). Subgroup analysis also showed that the effects of resveratrol on serum ALP were not significantly changed by the pathological conditions of the participants, including participants with diabetes (MD: 6.89, 95% CI: − 5.10, 18.89, *p* = 0.26) or without diabetes (MD: 1.61, 95% CI: − 3.88, 7.10, *p* = 0.57) (Fig. 5d), with NAFLD (MD: 10.34, 95% CI: − 13.69, 34.38, *p* = 0.40) or without NAFLD (MD: 2.17, 95% CI: − 2.93, 7.28, p = 0.40) (Fig. 5e), and with obesity (MD: 1.51, 95% CI: − 4.40, 7.42, *p* = 0.62) or without obesity (MD: 5.07, 95% CI: − 4.27, 14.40, *p* = 0.29) (Fig. 5f).

### Adverse events

Reporting of adverse events was limited, suggesting that resveratrol supplementation is well tolerated. Three RCTs reported the most frequent complaints were mild gastrointestinal symptoms including increased frequency of bowel motions and loose stools [23, 30, 36]. One study reported four adverse events, which were not necessarily attributable to the resveratrol supplementation [25]. In another study, one subject from the resveratrol group developed a transient pruritic skin rash, which resolved 14 days after having stop taking it [23]. One study reported that two patients in the resveratrol group developed serious adverse events: a case of gastrointestinal side-effects and a serious case of febrile leukopenia and thrombocytopenia after 10 days of resveratrol treatment [36]. The other studies reported that the rate of adverse events was low and the treatment was well tolerated [24, 37–40].

## Discussions

### Summary of main findings

The current meta-analysis aimed to evaluate the impact of resveratrol supplements on BMD and bone biomarkers compared with placebo. Here, we revealed that resveratrol supplementation compared with placebo did not significantly increase BMD at lumbar spine, total hip and whole body. In addition, resveratrol supplementation did not significantly change the expression of serum bone biomarkers including ALP, BAP, OCN, PINP, CTX and PTH. However, in view of the presence of some deficiencies among the included studies, such as the high risk of bias in some RCTs, the limited number of included studies and cases, and the clinical heterogeneity among the trials such as the dosage, intervention duration, and study population, the certainty of the current evidence is very low, which should be interpreted with caution.

### BMD of lumbar spine, total hip and whole body

Our findings are inconsistent with those of previous in vivo animal studies that suggested resveratrol supplementation increases BMD in aging, ovariectomy (OVX) and immobilization induced bone loss mouse models [12–14] and in vitro cellular studies that indicated resveratrol promotes osteoblast associated bone formation and inhibits osteoclast associated bone resorption [1, 51]. Such discrepancies between animal and human clinical studies are not unexpectable.

Firstly, results from in vitro studies should be interpreted with caution when trying to extrapolate the effect of resveratrol in vivo due to the influence of various complicated factors, including inter-species differences in terms of metabolism, absorption and tissue distribution, on the bioavailability of resveratrol in the target tissues [52, 53]. Secondly, the dose-dependent effect of resveratrol on bone quality is not adequately studied in clinical studies. Recent evidence of more effective outcome at lower resveratrol doses may imply the need to have more adequate pharmacological studies in preclinical settings to justify the selection of more appropriate dose for clinical trials [54, 55].. Another problem is blood concentrations of resveratrol are often too low to be detected due to rapid absorption and clearance from the body [56, 57], which limited the estimation of the effective doses of resveratrol in human. Development of novel delivery systems and resveratrol analogs with higher bioavailability [58] are of great interest in the future studies. It is noteworthy that vitamin D could amplify the bioavailability of resveratrol [59], thus it would be interesting in the future to investigate if the combination of resveratrol and vitamin D could further improve its effect on bone density via addition effects on mineral homeostasis and bioavailability enhancement. Thirdly, the intervention period of resveratrol is critical for the outcome of BMD. DXA scan is the most widely used procedure in the evaluation of BMD, however, BMD in human changes slowly with treatment thus the changes might not be detectable if the follow-up period is not adequately designed. Therefore, caution should be taken when interpreting the results of any treatment on BMD outcome with short intervention period [60]. In this review, two RCTs evaluating the outcome of BMD had follow-up time shorter than one year, both of which reporting negative results of areal BMD (aBMD) between resveratrol and placebo group after resveratrol supplementation [23, 24]. The study by Wong et al. with follow-up time more than one year reported that BMD significantly increased at lumbar spine by calculating the change from baseline between the placebo group and resveratrol group instead of the endpoint data [25]. It is noteworthy that in the study by Ornstrup et al., volumetric BMD (vBMD) of lumbar spine measured by quantitative computed tomography (QCT) showed significant increase after resveratrol supplementation whereas aBMD derived DXA showed no significant difference, suggesting QCT might be more sensitive to the treatment effects on BMD [23]. Future studies with longer follow-up time and QCT technique are needed to better elucidate the effects of resveratrol on BMD.

### Serum biomarkers

A previous meta-analysis of randomized controlled trials on the effects of resveratrol on bone biomarkers concluded that resveratrol supplementation increased serum level of ALP and BAP without changes in other bone turnover markers such as PINP, CTX, OCN, and PTH [26]. Our review included four more RCTs and found no significant effect of resveratrol supplementation on serum ALP. Possible reasons for the discrepancy could be the four newly included studies showing opposite results, with two showing unchanged serum ALP after resveratrol supplementation [24, 38], one showing significantly decreased ALP [39], and one showing significantly increased ALP level after resveratrol supplementation [40]. Another reason could be different types of data used in the meta-analysis. We used the endpoint data and the previous review used the change data from baseline. Nevertheless, it should be reminded that serum ALP is not a specific bone biomarker because it comes from several tissues including liver, kidney, intestine and bone [61]. Aberrant serum level of ALP is associated with diverse diseases such as cirrhosis, hepatitis, Paget’s disease and etc. apart from bone conditions [62]. On the other hand, BAP is the bone-specific isoenzyme of total ALP, which is considered as a more specific marker of osteoblastic activity. Our results showed a trend of increase in serum level of BAP following resveratrol supplementation, whereas the difference did not reach the statistical significance. OCN is a small non-collagenous protein hormone synthetized by osteoblast during the mineralization of matrix and is used as a preliminary biomarker on the effectiveness of a drug on bone formation [63]. We did not see any significant difference following resveratrol supplementation, which is consistent with the previous review [26]. PINP is cleaved from type I procollagen by osteoblast, which reflects the integrated amount of skeletal new bone formation [64]. It showed no significant difference after resveratrol supplementation compared to placebo. CTX is a telopeptide generated by collagen degradation and serve as a specific marker of bone resorption [65]. Our results revealed a non-significant reduction in serum CTX following the resveratrol supplementation. PTH plays a central role in the maintenance of calcium homeostasis through its effect on bone remodeling [66]. Our results showed a non-significant reduction in serum PTH after resveratrol supplementation. The effect of resveratrol on serum PTH level should be interpreted with caution because there were only two studies reporting such effect which is therefore vulnerable to sensitivity analysis.

These findings should be interpreted with caution due to the small number of studies and the potential heterogeneity among these studies such as methods and timepoint of the examination. In addition, single time-point examination of the bone turnover markers may not reflect the overall bone remodelling status because these bone biomarkers showed temporal changes after resveratrol supplementation [23]. Future studies are encouraged to examine the dynamic changes of the bone biomarkers following treatment.

### Limitations

There are several limitations in the current study. Firstly, there are some deficiencies in the methodological quality of the included studies. For example, the risk of bias of some RCTs are high; three of the included studies have not reported the final measurement (high risk of bias of incomplete outcome data) and some of the studies have unclear risk of bias of random sequence generation and allocation concealment, which could possibly lower the statistical power to detect the effect of intervention on the outcomes. The means and SD of the change from baseline are not available in most included studies (8 out of 10 studies) and it is difficult to compute the mean and SD because the baseline and final measurements are often reported for different numbers of participants due to missed visits and study withdrawals. Secondly, there are limited number of trials in the meta-analysis, which did not allow us to perform further subgroup meta-analysis of BMD and other bone markers except ALP with respect to the intervention period, dosage, or pathological conditions. Furthermore, the small number of included studies also makes it difficult to quantitatively examine the influences of clinical heterogeneity on our pooled results. For example, we could not examine the effect of age difference, sex difference, and compliance on the outcome. Thirdly, there is unaccountable clinical heterogeneity such as different doses of resveratrol ranging from 8 mg to 1000 mg, sample size ranging from 10 to 65, and different intervention periods ranging from 4 weeks to 12 months in our meta-analysis, potentially introducing bias to our analysis. Therefore, we performed the random effects analysis, which is better for the studies with potential heterogeneity. Lastly, some studies did not report sufficient data for meta-analysis, and several assumptions were made to impute missing SDs, further limiting the robustness of the meta-analysis. We are also concerned of the potential publication bias as only a few studies are available for each outcome and most of them have small sample size. Therefore, the certainty of current evidence is relatively low due to these limitations. All the results should be interpreted more cautiously.

Future trials with enough follow-up time and strong rationale as to the study population and doses of resveratrol are needed to accurately assess the effect of resveratrol supplementation on BMD and bone biomarkers, determine the suitable dose of resveratrol for particular population, whether accrue with increasing duration of supplementation, and whether benefits persist after supplementation ceases.

## Conclusions

In summary, we did not find any significant effects of resveratrol on BMD and bone biomarkers. However, the certainty of the current evidence is very low. More well-designed trials are needed to confirm whether long-term resveratrol supplementation might improve BMD and bone turnovers with reference to different durations, doses of resveratrol and study population.

## Supplementary Information

**Additional file 1: Table S1.** Summary of the searching strategy. **Table S2.** Sensitivity analysis of the effect of resveratrol on BMD of lumbar spine. **Table S3.** Sensitivity analysis of the effect of resveratrol on BMD of total hip. **Table S4.**. Sensitivity analysis of the effect of resveratrol on BMD of whole body. **Table S5.** Sensitivity analysis of the effect of resveratrol on serum ALP

## Data Availability

All data relevant to the study are included in the article or uploaded as supplementary information.

## Abbreviations

aBMD: areal bone mineral density
vBMD: volumetric bone mineral density
RCT: randomized controlled trials
MD: mean difference
ALP: alkaline phosphatase
BAP: bone alkaline phosphatase
OCN: osteocalcin
PINP: procollagen I N-terminal propeptide
CTX: C-terminal telopeptide of type I collagen
PTH: parathyroid hormone
MeSh: Medical Subject Headings
BMC: bone mineral content
OCN: osteocalcin
NTX: N-telopeptide of type I collagen
DXA: dual-energy X-ray absorptiometry
SD: standard deviation
SEM: standard error of the mean
CI: confidence interval
NAFLD: non-alcoholic fatty liver disease
OVX: ovariectomy
QCT: quantitative computed tomography
