# Supplementary material for: Effects of resveratrol supplementation on bone quality: a systematic review and meta-analysis of randomized controlled trials
Source: BMC Complement Med Ther. 2021 Aug 22;21:214. doi: 10.1186/s12906-021-03381-4 (PMC8380387; doi:10.1186/s12906-021-03381-4)
Supplement: Supplementary file 1 — Additional file 1: Table S1. Summary of the searching strategy. Table S2. Sensitivity analysis of the effect of resveratrol on BMD of lumbar spine. Table S3. Sensitivity analysis of the effect of resveratrol on BMD of total hip. Table S4.. Sensitivity analysis of the effect of resveratrol on BMD of whole body. Table S5. Sensitivity analysis of the effect of resveratrol on serum ALP [file 12906_2021_3381_MOESM1_ESM.zip › Supplementary table 2 3 4 5R4_0.docx]

Supplementary table 1 Summary of the searching strategy

Supplementary table 2 Sensitivity analysis of the effect of resveratrol on BMD of lumbar spine

| Study name | Statistics for each study | | | | |
| --- | --- | --- | --- | --- | --- |
|  | | Mean difference | 95% CI | Z-value | p-value |
| Ornstrup *et al*. 2014^(a)^ | | -0.02 | [-0.06, 0.03] | 0.72 | 0.47 |
| Ornstrup *et al*. 2014^(b)^ | | -0.03 | [-0.08, 0.02] | 1.22 | 0.22 |

BMD, bone mineral density; CI: confidence interval. The number

Supplementary table 3 Sensitivity analysis of the effect of resveratrol on BMD of total hip

| Study name | Statistics for each study | | | |
| --- | --- | --- | --- | --- |
|  | Mean difference | 95% CI | Z-value | p-value |
| Ornstrup *et al*. 2014^(a)^ | -0.01 | [-0.04, 0.03] | 0.38 | 0.71 |
| Ornstrup *et al*. 2014^(b)^ | -0.00 | [-0.03, 0.03] | 0.01 | 0.99 |

BMD, bone mineral density; CI: confidence interval.

Supplementary table 4 Sensitivity analysis of the effect of resveratrol on BMD of whole body

| Study name | Statistics for each study | | | | |
| --- | --- | --- | --- | --- | --- |
|  | | Mean difference | 95% CI | Z-value | p-value |
| Ornstrup *et al*. 2014^(a)^ | | 0.00 | [-0.02, 0.02] | 0.31 | 0.76 |
| Ornstrup *et al*. 2014^(b)^ | | 0.01 | [-0.01, 0.03] | 0.68 | 0.49 |

BMD, bone mineral density; CI: confidence interval.

Supplementary table 5 Sensitivity analysis of the effect of resveratrol on serum ALP

| Study name | Statistics for each study | | | |
| --- | --- | --- | --- | --- |
|  | Mean difference | 95% CI | Z-value | p-value |
| Anton *et al*. 2014^(a)^ | 2.84 | [-2.24, 7.92] | 1.10 | 0.27 |
| Anton *et al*. 2014^(b)^ | 2.59 | [-2.50, 7.68] | 1.00 | 0.32 |
| Asghari *et al*. 2018 | 2.43 | [-2.58, 7.44] | 0.95 | 0.34 |
| Heebøll *et al.* 2016 | 2.28 | [-2.81, 7.36] | 0.88 | 0.38 |
| Ornstrup *et al*. 2014^(a)^ | 3.93 | [-1.62, 9.49] | 1.39 | 0.17 |
| Ornstrup *et al*. 2014^(b)^ | 3.14 | [-2.40, 8.67] | 1.11 | 0.27 |

ALP: alkaline phosphatase; CI: confidence interval.
